# Polymorphisms of the Chicken Mx Gene Promoter and Association with Chicken Embryos' Susceptibility to Virulent Newcastle Disease Virus Challenge

**DOI:** 10.1155/2019/1486072

**Published:** 2019-10-03

**Authors:** Fulgence N. Mpenda, Christian T. Keambou, Martina Kyallo, Roger Pelle, Sylvester L. Lyantagaye, Joram Buza

**Affiliations:** ^1^School of Life Sciences and Bioengineering, Nelson Mandela African Institution of Science and Technology, P.O. Box 447, Tengeru, Arusha, Tanzania; ^2^Biosciences Eastern and Central Africa, International Livestock Research Institute, Nairobi, Kenya; ^3^Department of Molecular Biology and Biotechnology College of Natural and Applied Sciences, University of Dar es Salaam, Dar es Salaam, Tanzania

## Abstract

Newcastle disease is a devastating viral disease of chicken in low- and middle-income countries where the backyard production system is predominant. Marker-assisted selection of chickens that are resistant to Newcastle disease virus (NDV) is the promising strategy that needs to be explored. The aim of the present study was to investigate polymorphisms of the promoter region of the chicken Mx gene and association with Kuroiler, Sasso, and local Tanzanian chicken embryos' survival variability to virulent NDV infection. Chicken embryos were initially challenged with a minimum lethal dose of virulent NDV suspension and then were followed over time to gather information on their survival variability. Using the survival data, high and less susceptible cohorts were established, and a total of 88 DNA samples from high and less susceptible groups were genotypes by sequencing. Five single-nucleotide polymorphisms (SNPs), which were previously reported, were detected. Interestingly, for the first time, the findings demonstrated the association of the promoter region of chicken myxovirus-resistance (Mx) gene polymorphisms with chicken embryos' susceptibility to the virulent NDV challenge. At the genotypic level, the SNP4 *G* > *A* mutation that was located within the IFN-stimulating response element was associated (LR: 6.97, *P*=0.03) with chicken embryos' susceptibility to the virulent NDV challenge. An allele *G* frequency was higher in the less susceptible cohort, whereas an allele *A* frequency was higher in the high susceptible cohort. At the haplotype level, the haplotype group ACGC was associated (OR: 9.8, 95% CI: 1.06–79.43, *P*=0.042) with the same trait and had a resistant effect. In conclusion, the results have demonstrated the association of chicken Mx gene promoter polymorphisms and chicken embryos' survival variability to the virulent NDV challenge, and the information is useful for breeding programs designed to develop chicken genotypes that are resistant to Newcastle disease virus.

## 1. Introduction

The myxovirus-resistance (Mx) genes are found in a wide range of living organisms including chicken [1]. Mx proteins are interferon- (IFN-) induced GTPase enzymes with antiviral functions, which particularly play a significant role in inhibition of negative-stranded RNA viruses [1, 2]. Binding of type I or III IFNs to receptors triggers the expression of IFN-stimulated genes, thereby inducing an antiviral state within a cell. Therefore, the expression of Mx genes largely depends on the activation of type I or III IFNs [1, 3].

The genomic size of the chicken Mx gene is about 21 kbps with the coding sequences of about 2118 bps encoding the Mx protein comprising 705 amino acids [4, 5]. Like other IFN-response genes, the chicken Mx gene contains a sequence element in its promoters that serve as inducible enhancers. The chicken Mx gene promoter contains a motif 5′-AGGTTTCTTTCCT-3′ or its reverse complement [6, 7], which is an integral part of the IFN-stimulated response element (ISRE). It has been documented that the ISRE motif has a crucial role to play in IFN inducibility of the chicken Mx gene [6].

Multiple allelic variants of the chicken Mx gene have been reported in different populations of chicken throughout the world [4, 8, 9]. For example, Li et al. [4] reported a total of 24 single-nucleotide polymorphisms (SNPs) after comparison of four chicken sequences. The highest nucleotide diversity (*π* value: 0.01003) was in the chicken Mx gene promoter where a total of six SNPs were found [4]. A similar finding was observed when nine elite egg-layer-type lines were sequenced where a total of 6 SNPs out of 36 SNPs that were reported were found in the chicken Mx gene promoter [9].

Most of the available reports have demonstrated the role of the chicken Mx gene G2032A (S631N) in antiviral activities, particularly against influenza virus [10–12]; however, a considerable number of reports failed to demonstrate the same association [8, 13]. These conflicting reports may suggest that the antiviral activity of the chicken Mx gene involves several allelic variants, and more remain to be elucidated. Therefore, the present study was aimed to investigate the polymorphisms of the chicken Mx gene promoter and association with Kuroiler, Sasso, and local Tanzanian chicken embryos' survival variability to the virulent NDV challenge.

## 2. Materials and Methods

### 2.1. Source of the Virulent Newcastle Disease Virus (NDV) Isolate

A virulent NDV field isolate from live birds was kindly provided by Sokoine University of Agriculture. Characterization was done as previously described [14–17] to confirm virulence of the strain before the experiment was conducted. The virus was then titrated to a working titre of a minimum lethal dose (MLD) of 10^3^/0.1 mL of virus suspension, and the viral suspension was stored at −80°C until use.

### 2.2. Embryonated Chicken Eggs' Challenge

The experiment involved three chicken types, which had the same history of NDV immunization: Tanzanian local chicken, Kuroiler [18], and Sasso [19]. Chicken embryos' variability in susceptibility to virulent NDV infection was evaluated by detection of chicken embryo survival time following infection with the MLD of the viral suspension. Therefore, sixteen-day-old embryonated eggs were inoculated with 0.1 ml (10^3^ MLD/0.1 mL) of virus suspension directly deposited into the allantoic fluid by using a 1 ml sterile syringe. Eggs were sealed with adhesive glue and incubated at 37.9°C for a total of 120 hours after infection (pi). During this time, except for first 24 hours after infection, embryonated eggs were candled at the time interval of 6 hours to detect dead embryos. Dead embryonated eggs were chilled at 4°C for four hours before tissue harvest. In the experiment, a total of 355 (87 Sasso, 129 Kuroiler, and 139 local chicken) embryonated eggs were challenged in three experimental replicates.

### 2.3. Chicken Embryo Tissue Harvest and DNA Extraction

In a biological safety cabinet, dead embryonated eggs were decontaminated by using 70% ethanol. Then, leg muscles and comb were harvested and stored in separate tubes, which were then stored at −20°C for genomic DNA extraction. The selective genotyping approach was employed as it was previously demonstrated [20, 21]. Thus, using chicken embryos' survival data, genomic DNA was extracted from high (15%) and less (15%) susceptible groups. Genomic DNA was extracted from comb or leg tissues using the Quick-DNA Tissue/Insect Kit (Zymo Research) in accordance with the manufacturer's instructions. The quality and quantity of genomic DNA were assessed by running on 1% (w/v) agarose gel containing ethidium bromide in 0.5% TBE buffer for an hour.

### 2.4. PCR Amplification

The polymerase chain reaction (PCR) was conducted to amplify a DNA fragment of about 284 bp region of the 5′ untranslated region and partial promoter of the chicken Mx gene [4]. The selected primers (forward primer: 5′-ACCTGTGCCATCTGCCCTCTGA-3′ and reverse primer: 5′-CACAGCAAGGAGAAACAATTAACTACAT-3′) and PCR conditions were as previously described [22]. The amplification was conducted in a reaction volume of 25 *μ*L containing 0.2 *μ*M of each primer and 12.5 *μ*L of Taq 2X PCR MasterMix (New England Biolabs (NEB)). The PCR conditions were as follows: initial denaturation at 94°C for 5 minutes, followed by 40 cycles of denaturation at 94°C for 1 minute, annealing at 58.5°C for 30 seconds, extension at 72°C for 1 minute, and final extension at 72°C for 5 minutes. The PCR ran on the QuantStudio 6 Flex Real-Time (RT) PCR Thermal Cycler (Applied Biosystems). The quality of PCR products was evaluated by running on 1% (w/v) agarose gel containing ethidium bromide in 0.5X TBE buffer at 100 V for an hour.

### 2.5. Sequencing and Bioinformatics Analysis

Raw sequences were trimmed using CLC Genomics Workbench v3.0.8 and consensus sequences generated. A total of 88 sequences (24 Kuroiler, 32 local chicken, and 32 Sasso) were further analyzed. Multiple-sequence alignment was done using the MUSCLE algorithm in MEGA v6 to identify polymorphic sites. SNP calling was done using CLC Genomics Workbench v3.0.8.

### 2.6. Statistical Analysis

Population genetics parameters like the Hardy–Weinberg equilibrium, pairwise linkage disequilibrium, and association analysis were performed by using SNPStats, a web tool for SNP analysis [23]. Other statistic tests like Pearson's chi-squared test of independence of genotype frequencies and likelihood ratio tests were conducted by using the R software (version 3.3.3; The R Foundation for Statistical Computing).

## 3. Results

### 3.1. Chicken Embryos' Survival Time after Challenge with Virulent NDV

**Figure 1:**
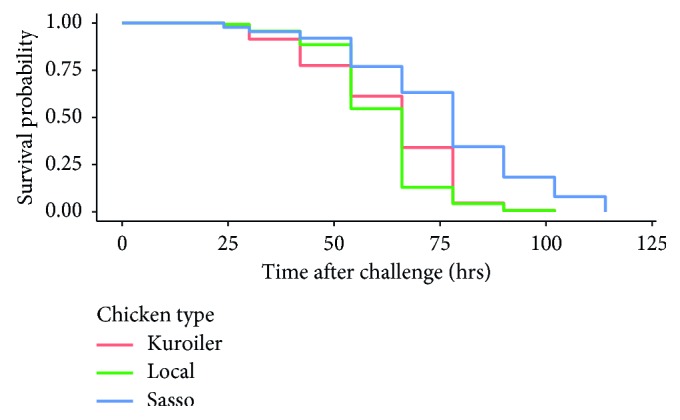
Kaplan–Meier survival curves of chicken embryos following the challenge with virulent Newcastle disease virus observed in the present study.

The survival time probability of developing chicken embryos upon challenge with virulent NDV is presented in Figure 1. Overall, Sasso chicken embryos had the highest survival time probability as compared to Kuroiler and local Tanzanian chicken embryos (Figure 1).

### 3.2. Sequence Variations in the Amplified Promoter Region of the Chicken Mx Gene

A total of five single-nucleotide polymorphisms (SNPs) were observed in the present study (Table 1). All SNPs were previously reported when the same (284 bp) promoter region of the chicken Mx gene was sequenced [22]. Generally, the observed and expected heterozygosity for all SNPs was at the same levels (Table 1). Furthermore, all the SNPs had no interaction between the response variable (susceptibility) and the covariate (breed), with the exception of SNP3. Additionally, SNP3 was not associated with chicken embryos' variability in susceptibility to virulent NDV infection, and therefore, SNP3 was removed from further analysis to allow breed merging.

### 3.3. The Hardy–Weinberg Equilibrium

The 5 SNPs were tested for agreement with the Hardy–Weinberg equilibrium (HWE). All SNPs were in consistence with the Hardy–Weinberg principle (*P* > 0.05) (Table 2). Also, the minor allele frequencies in high (H) and less (L) susceptible cohorts were greater than 0.05 (Table 2).

### 3.4. SNP Allele and Genotype Frequency

The allele and genotype frequencies of 4 SNPs (SNP1, SNP2, SNP4, and SNP5) are presented in Tables 2 and 3. At the allelic level, the results indicated that there was not association between SNPs and chicken embryos' susceptibility to the virulent NDV challenge. However, at the genotypic level, SNP4 (LR = 6.97, *P* < 0.05) was significantly associated with chicken embryos' susceptibility to virulent NDV infection (Table 3).

### 3.5. Linkage Disequilibrium and Haplotype Frequency

Results of linkage disequilibrium (LD) of 5 SNPs are shown in Table 4. All the SNPs were in LD (*P* < 0.05) with the exception of SNP3 (*P* > 0.05), which was in equilibrium with other SNPs (Table 4).

Haplotype analysis of four SNPs that were in LD generated 4 haplotypes (Table 5). The haplotype group “*ACGT*” had highest haplotype frequency (0.74), and the lowest haplotype frequency (0.01) was observed in the haplotype group “*AGGT*.” The haplotype group “*ACGC*,” which had a frequency of 0.06, was associated (*P* < 0.05) with chicken embryos' susceptibility to virulent NDV infection (Table 5).

## 4. Discussion

In the present study, a combination of candidate gene and selective genotyping approaches were employed to investigate polymorphisms of the promoter region of the chicken Mx gene and association with chicken embryos' susceptibility to virulent NDV infection. Chicken embryos were initially challenged with the MLD of virulent NDV suspension, and information was continuously gathered on their survival variability pi. Using the survival data, high and less susceptible cohorts were established for the promoter region of chicken Mx gene genotyping by sequencing. We were able to detect 5 SNPs, which had been reported previously by Mishra et al. [22].

Interestingly, for the first time, the findings demonstrated the association of the promoter region of chicken Mx gene polymorphisms with chicken embryos' susceptibility to virulent NDV infection. At the genotypic level, SNP4 was associated (*P* < 0.05) with the phenotype, whereas at the haplotype level, the haplotype group *ACGC* was associated (*P* < 0.05) with the phenotype. The SNP4 *G* > *A* mutation was detected at the 194^th^ position (Table 1) corresponding to 1 to 284 positions along the amplified product length polymorphisms for 5′ to 3′. SNP4 was within the AGTTTCGTTTCT motif of the ISRE, and the SNP was previously reported in the same position [4]. The ISRE plays a crucial role in IFN inducibility of the chicken Mx gene [6]. The results of the present study demonstrated the association (LR: 6.97, *P*=0.03) of the genotype *GG* of SNP4 with less susceptibility of chicken embryos to virulent NDV infection. The genotype *GG* frequency of 0.7 in the less susceptible group was higher as compared to the genotype *GG* frequency of 0.63 in the high susceptible group. Likewise, although it was not statistically significant, allele *G* had a higher frequency (0.85) in the less susceptible group as compared with the allele *G* frequency (0.76) in the high susceptible group. Also, allele *A* had high frequency (0.24) in the high susceptible group as compared with the allele *A* frequency (0.15) in the less susceptible group. It can be said that the mutation of *G* > *A* is associated with susceptibility of chicken embryos to virulent NDV infection, whereas allele *G* is associated with resistance of the same trait. The mechanisms underlying this observation remain to be elucidated; however, it may be that the SNP4 *G* > *A* mutation results diminished the role of the ISRE in IFN inducibility of the chicken Mx gene, thereby resulting in less expression of the gene, which has been demonstrated to play a role in antiviral function.

Interaction of SNPs can generate the same phenotypic information that can be obtained by individual SNP [24]. Analysis of SNP haplotypes is effective and less expensive. Indeed, in the present study, four haplotypes were generated using SNPs that were in LD (Table 4) and were tested for association with chicken embryos' susceptibility to virulent NDV infection. The haplotype group ACGC was significantly (OR: 9.8, 95% CI: 1.06–79.43, *P*=0.042) associated with chicken embryos' susceptibility to virulent NDV infection. The frequency (0.1) of the haplotype group ACGC was high in the less susceptible group compared to the high susceptible group, which had a frequency of 0.01. The haplotype group was demonstrated to have the protective effect upon chicken embryo infection with virulent NDV.

Taken together, the polymorphisms of the chicken Mx gene promoter are associated with chicken embryos' variation in susceptibility to virulent NDV infection. The SNP4 *G* > *A* mutation at the 194^th^ position of the amplified region (284 bp) was associated with the phenotype. More importantly, the haplotype group ACGC generated by the combination of SNP1, SNP2, SNP4, and SNP5 was demonstrated to have the resistant effect on chicken embryos infected with virulent NDV. The information is useful for breeding programs designed to develop chicken genotypes that are resistant to Newcastle disease virus.

## Figures and Tables

**Table 1:** Single-nucleotide polymorphic sites observed in the study.

Markers	Position	*H*_O_	*H*_E_	Allele change
SNP1	91^st^	0.34	0.28	*A* > *T*
SNP2	107^th^	0.34	0.32	*C* > *G*
SNP3	113^th^	0.34	0.38	*C* > *T*
SNP4	194^th^	0.28	0.30	*G* > *A*
SNP5	231^st^	0.31	0.37	*T* > *C*

*H*_O_: observed heterozygosity; *H*_E_: expected heterozygosity. Position is the polymorphic site corresponding to 1 to 284 positions along the amplified product length from 5′ to 3′.

**Table 2:** Allele frequency of polymorphic sites of the promoter region of the chicken Mx gene and association with chicken embryos' susceptibility to virulent NDV infection.

Markers	Allele	Susceptibility		*P* value (*χ*^2^)	*P* value (LR)	*P* value (HWE)	MAF
		H	L				
SNP1	*A*	59 (0.78)	85 (0.85)	0.02	0.01	1	0.16
	*T*	17 (0.22)	15 (0.15)	0.89	0.09		

SNP2	*C*	58 (0.76)	84 (0.84)	1.64	1.62	1	0.20
	*G*	18 (0.24)	16 (0.16)	0.20	0.2		

SNP4	*G*	58 (0.76)	85 (0.85)	2.14	2.12	0.49	0.19
	*A*	18 (0.24)	15 (0.15)	0.14	0.14		

SNP5	*T*	58 (0.76)	75 (0.75)	0.04	0.04	0.14	0.24
	*C*	18 (0.24)	25 (0.25)	0.84	0.84		

*χ* ^2^, Pearson's chi-squared test of independence; LR, Likelihood ratio; HWE, Hardy–Weinberg equilibrium test; H, high susceptible chicken embryo group; L, less susceptible chicken embryo group; MAF, minor allele frequency.

**Table 3:** Genotype frequency of polymorphic sites of the chicken Mx gene promoter and association with chicken embryos' susceptibility to virulent NDV infection.

Markers	Genotype	Susceptibility		*P* value (*χ*^2^)	*P* value (LR)
		H	L		
SNP1	*AA*	24 (0.63)	35 (0.7)	4.11	5.20
	*AT*	11 (0.29)	15 (0.3)	0.13	0.07
	*TT*	3 (0.08)	—		
SNP2	*CC*	23 (0.61)	34 (0.68)	4.13	5.23
	*CG*	12 (0.32)	16 (0.32)	0.13	0.07
	*GG*	3 (0.08)	—		
SNP4	*AA*	4 (0.11)	—	5.52	6.97
	*AG*	10 (0.26)	15 (0.3)	0.04	0.03
	*GG*	24 (0.63)	35 (0.7)		
SNP5	*CC*	4 (0.11)	4 (0.08)	0.67	0.67
	*CT*	10 (0.26)	17 (0.34)	0.72	0.72
	*TT*	24 (0.63)	29 (0.58)		

*χ* ^2^, Pearson's chi-squared test of independence; LR, likelihood ratio.

**Table 4:** The linkage disequilibrium *r* statistic for five SNPs reported in the present study.

	SNP1	SNP2	SNP3	SNP4	SNP5
SNP1	—	0.96	−0.28	0.98	0.83
SNP2	—	—	−0.29	0.94	0.79
SNP3	—	—	—	−0.28	0.07
SNP4	—	—	—	—	0.81
SNP5	—	—	—	—	—

**Table 5:** Haplotype analysis of four polymorphic sites of the chicken Mx gene promoter that are in LD and association with chicken embryos' susceptibility to virulent NDV infection.

No.	Haplotypes				Frequencies			OR (95% CI)	*P* value
	SNP1	SNP2	SNP4	SNP5	Total	H	L		
1	*A*	*C*	*G*	*T*	0.74	0.74	0.74	1.00	—
2	*T*	*G*	*A*	*C*	0.06	0.22	0.15	0.54 (0.21–1.36)	0.2
3	*A*	*C*	*G*	*C*	0.06	0.01	0.10	9.18 (1.06–79.43)	0.042
4	*A*	*G*	*G*	*T*	0.01	0.01	0.01	0.73 (0.04–12.88)	0.83

CI, confidence interval; OR, odds ratio; H, high susceptible chicken embryo group; L, less susceptible chicken embryo group.

## Data Availability

The data used to support the findings of this study are available from the corresponding author upon request.
